# Porcine ear necrosis in weaned piglets: prevalence and impact on daily weight gain

**DOI:** 10.1186/s40813-021-00240-z

**Published:** 2021-12-13

**Authors:** Mateusz Malik, Alexandra Schoos, Ilias Chantziaras, Dries Donkers, Siska Croubels, Barbara Doupovec, Dominiek Maes

**Affiliations:** 1 Department of Internal Medicine, Reproduction and Population Medicine, Faculty of Veterinary Medicine, Ghent University, Ghent, Belgium; 2 Department of Pathobiology, Pharmacology and Zoological Medicine; Faculty of Veterinary Medicine, Ghent University, Ghent, Belgium; 3 Biomin Research Center, Technopark 1, Tulln, Austria

**Keywords:** Ear necrosis, Weaned pigs, Prevalence, Average daily gain

## Abstract

**Background:**

Porcine ear necrosis (PEN) in pigs is characterized by a blue to black discoloration of the tip or margin of the ear followed by necrosis. The present study investigated the prevalence of PEN in a Belgian pig farm with PEN problems in nursery pigs, the effect of a mycotoxin detoxifier added to the feed on PEN prevalence, and the impact of PEN on the piglets’ growth. Six consecutive batches of weaned piglets [565–751 piglets per batch, (n = 3898)] were included. For each weaning batch, the presence and severity of PEN during the nursery period (3–10 weeks of age) were recorded weekly. Average daily gain (ADG) was calculated by weighing 597 individual piglets divided over the six batches. Additionally different mycotoxins were measured in the feed using LC–MS/MS analysis, and to three randomly selected batches, a mycotoxin detoxifier (Mycofix® Plus 5E, Biomin) was added to the feed.

**Results:**

At the end of the nursery period, 11.0–32.0% of the piglets in each batch were affected. The prevalence increased with the number of weeks post-weaning, especially from week 4 after weaning onwards. Mild, moderate, severe and very severe lesions represented 84.6%, 14.0%, 1.3% and 0.1% of all lesions, respectively. Different mycotoxins were present in the feed, but all at low concentrations. The mean ADG (± SD) for pigs without (n = 243) and with (n = 158) lesions was 391 g (± 71 g) and 394 g (± 65 g), respectively (*P* > 0.05). The ADG for mildly affected (387 g ± 68 g) and moderately affected piglets (420 g ± 44 g) was not significantly different (*P* > 0.05). The PEN prevalence in the batches without or with the mycotoxin detoxifier was 25% and 22%, respectively (*P* > 0.05).

**Conclusions:**

Twenty-three percent of animals showed lesions at the end of the nursery. Affected pigs did not have a lower ADG compared to non-affected animals, which might be explained by the fact that most affected piglets only had mild lesions. The addition of a mycotoxin detoxifier did not influence the prevalence of PEN, possibly because of the low levels of mycotoxin contamination. Further research is warranted to assess the impact of more severe PEN lesions and the effect of control measures.

## Background

Porcine ear necrosis (PEN) is characterized by blue to black discoloration of the ear tip or margin followed by necrosis. The lesions may vary in severity and mainly occur in weaning pigs. The condition has been described already in the eighties [1] but so far, the etiology nor the pathogenesis are known, and it has not yet been possible to experimentally reproduce PEN. In general, three hypotheses for the disease development have been put forward [2]. A first hypothesis states that the necrosis starts on the outer surface of the injured skin and is caused by toxins of *Staphylococci* [3]. A second hypothesis states that PEN is due to damage or occlusion of small blood vessels caused e.g. by cold agglutinins or immune complexes which can be produced during infection with specific pathogens such as *Mycoplasma suis* [4]. A third hypothesis states that external trauma e.g. due to biting on the ear or environmental factors is causing the PEN problems [3].

Different potential risk factors triggering lesion formation have been reported in literature, such as poor air quality, high humidity, low availability of drinkers or feeders, high stocking density, and contamination of the feed with mycotoxins [3, 5]. Among these factors, also Microbe-Associated Molecular Patterns (MAMPs) which include molecules like lipopolysaccharide, flagellin, lipoteichoic or nucleic acid variants [6], have been proposed as a possible trigger of local inflammatory processes in blood vessels, leading to necrosis. [7].

If considering biting as a cause of PEN, the work of Nordgreen et al. [8]. hypothesizes how different factors such as infections, housing conditions, stress, gut microbiota, and amino acid composition or mycotoxin contamination of the feed, may affect the behavior of the pigs via pro-inflammatory cytokines.

Although there is no evidence of direct involvement of mycotoxins in PEN yet, mycotoxins such as trichothecenes are considered as a risk factor because of their potential immunosuppressive and dermonecrotic effects [9] as well as tissue necrosis via vasoconstriction due to ergot alkaloids [10]. Several studies [11–13] have suggested a potential role of mycotoxins in the development of PEN lesions.

In order to better understand PEN and to optimize control measures, it is important to have more precise information on the prevalence and severity of PEN as well as on the impact of PEN on performance. Information about these aspects is however scarce in literature. Previous studies showed that the prevalence of PEN may exceed even 80%, and varies largely between different farms and studies [14–17]. Papatsiros [18] mentioned that lesions started to appear in 5–6 week old piglets, reaching a prevalence of approximately 20%, and that affected piglets suffered from poor growth and health problems later on. The study of Park [3], showed that lesions started to occur at seventh week of age, and generally increased in severity over a period of approximately 4 weeks. Busch et al. [14] reported that 70% of the pigs were affected 1 week after weaning, with a decrease to 20% 7 weeks later. PEN had no effect on the pigs growth, yet no data were given. In a subsequent study of the same research group, PEN occurred already 1 day after weaning, and the prevalence quickly increased to 45% 3 days later [19]. As only few and contradictory results are reported in literature, more research on PEN is warranted.

To the authors’ knowledge, there are no studies that have assessed the evolution of PEN in a large number of pigs from successive batches, the impact of mycotoxin detoxifiers and measured the impact of PEN on piglets growth. The present study investigated the prevalence and severity of PEN in a Belgian pig farm with PEN problems in nursery pigs, the effect of a mycotoxin detoxifier added to the feed on PEN prevalence and the impact of PEN on the piglets’ growth. To this end, six consecutive batches of weaned pigs were monitored throughout the entire nursery period.

## Materials and methods

The experimental study protocol was approved by the Ethical Committee of the Faculty of Veterinary Medicine and the Faculty of Bioscience Engineering, Ghent University (EC2019-71), as well as by the Flemish governmental agency for animal welfare (DWZ/ER/19/1.15/72).

### Herd management and housing of the animals

The study was conducted on a Belgian farrow to-finish farm with PEN problems in the nursery unit. The farm had 300 Danbred sows, 1300 nursery pigs and approximately 2500 fattening pigs. The farm practiced a 4-week batch production system and piglets were weaned at 3 weeks of age. At 4–5 days of age, piglets received an iron injection (Ferraject®, 200 mg, Dechra, Bladel, Netherlands) intramuscularly and toltrazuril (Baycox®, Bayer, Leverkusen, Germany,) against *Cystoisospora suis* infections, orally. Male piglets were not surgically castrated, but injected with a gonadotropin releasing hormone analogue (Improvac®, Zoetis, Zaventem, Belgium) in the fattening unit. At 3 weeks of age, the piglets were weaned, sorted by gender, and moved to the nursery unit where they stayed for 7 weeks, until 10 weeks of age. Within the first week after weaning, all weaners were vaccinated against *Mycoplasma hyopneumoniae* (*M. hyopneumoniae*) and Porcine Cirvovirus 2 (PCV2) (Porcilis PCV M Hyo®, MSD Animal Health, Boxmeer, Netherlands). Sows were not vaccinated against these pathogens. Because the farm had experienced problems in the nursery (locomotion and respiratory clinical signs), pigs were orally medicated after weaning with amoxycillin (Amoxy Active®, Dopharma, Raamsdonksveer, Netherlands) and if needed, individual animals were treated by intramuscular injection with amoxycillin (Vetrimoxin®, Ceva, Libourne, France).

The farm had two nursery stables with a capacity of 750 piglets each. Stables were divided into 56 pens in total. One pen included on average 28 piglets, corresponding to approximately 0.3 m^2^ of floor surface area per piglet. The compartments in stable 1 and 2 had a partially slatted floor, and heating was used in the solid part. In stable 1, the slatted part consisted of polyvinyl chloride (PVC) and the solid part of concrete. In stable 2, in some compartments, the slatted and the solid part consisted of PVC, in other compartments, metal slats were used in combination with a solid concrete part. Compartments were used according to the all-in/all-out principle, and they were cleaned and disinfected between each weaning batch. There was mechanical ventilation in both stables. Stable 1 had a door ventilation system with fans, and stable 2 had ceiling ventilation. At the beginning of the nursery period, the ambient temperature was set at 27 °C and then gradually decreased to 25 °C 4 weeks later.

Each nursery stable had its own feed silo and feeding line. Piglets in the nursery unit received feed and water ad libitum. Throughout the nursery period, three different types of feed were used. Transitions from one feed to another feed were done gradually during a period of 3–5 days. There was one dry feed trough per pen, with six feeding places each. Opposite to the feeder, there were three drinking nipples with water from a deep pit (120 m). A piece of wood on a chain, hay, and paper were used as environmental enrichment for the piglets in the nursery.

### Study population and general study design

The study was conducted from October 2019 to April 2020, and included six consecutive weaning batches with 560–750 piglets per batch. The pigs were monitored until the end of the nursery period i.e. until they were approximately 10 weeks of age. To three randomly selected weaning batches (2, 3, and 6), a mycotoxin detoxifier (Mycofix® Plus 5E, Biomin Holding GmbH, Getzersdorf, Austria; 2 kg/ton of feed) was administered to the commercial feed (meal) during the entire nursery period. The three other batches (1, 4 and 5) received the same feed without the detoxifier. This was done to assess a possible influence of the mycotoxin detoxifier on PEN in this farm.

### Data collection and sampling of entire weaning batch

The prevalence of PEN and severity of the lesions were assessed weekly by the same person in all animals and recorded at pen level. To this end, the person was observing the pigs from each individual pen. The observer was not aware in which batch the detoxifier was used. A five point scoring system was used to assess the severity of the lesions: score 0 = no deviations, score 1 = incipient red discoloration or a crust at the tip of the ear, score 2 = more black-like discoloration and a rounded ear tip, score 3 = severe necrosis with a part less than one third of the ear missing at the tip, and score 4 = piglets which lost more than one third of the ear or showed an equally large necrotic area [20]. At the end of each week, the number of pigs affected with ear necrosis was counted and divided by the total number of pigs present in the pen.

Mortality was calculated for each weaning batch upon completion of the nursery period.

Feed samples (n = 37) were taken from every feed delivery. There were six deliveries per batch, except for weaning batch 4 where seven deliveries took place. Each sample was collected by pooling feed from different (six to eight) feeding troughs throughout the stable and stored at room temperature. All feed samples were sent to IFA Department (Tulln, Austria), pooled by two (except for batch 4, for which 3 samples were pooled once) to be analyzed with a multi-mycotoxin LC–MS/MS analysis method (Spectrum 380®) for the presence of mycotoxins and metabolites [21].

Two water samples were collected from the drinking nipple of the last batch. The water samples were analyzed bacteriologically and chemically in the laboratory of Animal Health Care Flanders (Torhout, Belgium).

In weaning batches 4–6, the relative humidity (%) and temperature (°C) were recorded every 30 min using a Tinytag Plus2 logger. In each stable one logger was placed at a height of 1.2 m, in the middle of a compartment where the selected animals were housed.

### Data collection and sampling of a group from a weaning batch

From each batch, four pens with 50 gilts and 50 boars in total were randomly selected at the beginning of the nursery period. These pigs were housed in four neighbouring pens within one compartment in either stable 1 or 2. These piglets received an additional eartag and were weighed individually at the beginning and at the end of the nursery period. Selected pigs of the following batches were housed in the same compartments. Average daily gain was calculated by dividing the weight gain by the total number of days spent by the group in the entire nursery unit. Pigs which died during that period were not included in the calculation.

The lesion scores from weaning batches 3–6 were recorded at an individual animal level.

**Fig. 1:**
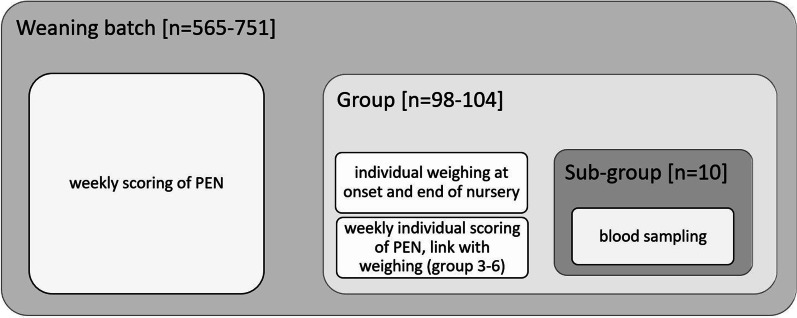
Number of pigs (n) and associated measurements or actions at the level of weaning batch, group within weaning batch and subgroup for blood sampling in all consecutive batches

From each group of 100 piglets, 10 animals were randomly selected and blood sampled at 4 and 7 weeks after weaning (Fig. 1). Blood samples were taken by puncture of the jugular vein, on serum and plasma tubes with EDTA. Blood samples were centrifuged for 10 min at 4 °C on 3725×*g* to obtain plasma, and subsequently stored at − 20 °C until further analysis. All plasma samples were analyzed using a validated multi-mycotoxin LC–MS/MS method to assess the presence of mycotoxins and relevant phase I and II metabolites [22].^1^

Blood samples were centrifuged for 10 min at 4 °C on 3725×*g* and sera were analyzed by hemagglutination inhibition test for Swine influenza (SI) type H1N1, H1N2, H3N2, and by ELISA for the presence of antibodies against PCV2 (IgM and IgG), porcine reproductive and respiratory syndrome virus (PRRSv) and *M. hyopneumoniae*. The serum samples were also tested by PCR to investigate the presence of PCV2 and PRRSv, the European and American strain. Serum samples were pooled by 4 for the PCR tests, but samples of PEN positive or PEN negative pigs were not mixed.

### Statistical analysis

Statistical analysis was performed using IBM® SPSS® Statistics for Windows Version 24 (IBM Corp., Armonk, N.Y., USA). Descriptive information for the aforementioned parameters is provided for the piglets. The information is categorized according to whether the piglets were affected by PEN and/or whether the piglets were fed with mycotoxin detoxifier or not. To statistically evaluate the associations between average daily gain (ADG) and the prevalence of PEN, analysis of variance (initial step) and a multivariable linear mixed regression model (final step) were used. The assumptions of normality and homogeneity of variance were tested by examining histograms, residual plots and plots of studentized residuals versus the predicted values. Additionally, the Kolmogorov Smirnov test was used to assess normality per group. In the final model, batch and pen were inserted as random factors and the use or not of the mycotoxin detoxifier was also inserted as a fixed factor. Average daily gain was considered as dependent variable. To evaluate the differences in the prevalence of PEN between the piglets that received the mycotoxin detoxifier-supplemented feed or not, a chi square test was used. To assess the repeatability of PEN prevalence, the interclass correlation coefficient was measured and statistically evaluated. For this calculation, only pens where pigs had been housed of three weaning batches, were included.

## Results

### Prevalence of PEN lesions

The weekly prevalence of PEN is shown in Table 1. At weaning, no animals showed signs of PEN. The prevalence of PEN tended to increase with the number of weeks post-weaning in each batch, especially from week 4 onwards. The highest prevalence at the end of the nursery was 31.6% and 32.0%, in batch 2 and 6, respectively. The lowest prevalence was observed in batch 3 (11.1%). In general, the highest prevalence was observed during the last week of the nursery, with one exception in batch 1, where the highest prevalence occurred in week 6 (23.8%) and slightly decreased 1 week later (21.8%).

**Table 1:** Weekly prevalence (%) of porcine ear necrosis

Weaning batch	1	2^a^	3^a^	4	5	6^a^	All batches
Number of piglets at weaning	588	565	687	678	751	629	3898
Week post-weaning	Prevalence (%)						Average
1	0.0	0.2	0.1	0.0	0.3	0.2	0.2
2	0.3	0.9	0.1	0.9	0.8	0.3	0.6
3	0.9	2.0	0.7	0.9	0.7	0.3	0.9
4	1.9	10.1	4.7	4.6	4.0	4.8	4.9
5	12.5	20.3	7.2	10.2	9.3	17.7	12.5
6	23.8	23.6	10.7	16.8	14.2	28.8	19.2
7	21.8	31.6	11.1	23.7	20.0	32.0	22.9
Total	61.2	88.7	34.6	57.1	49.3	84.1	38.3

Prevalence of PEN in weaned pigs during the nursery period. Values indicate the relation between affected piglets and the total number of pigs at the end of each week for each weaning batch

^a^A mycotoxin detoxifier was added to the feed of weaning batches 2, 3 and 6

**Fig. 2:**
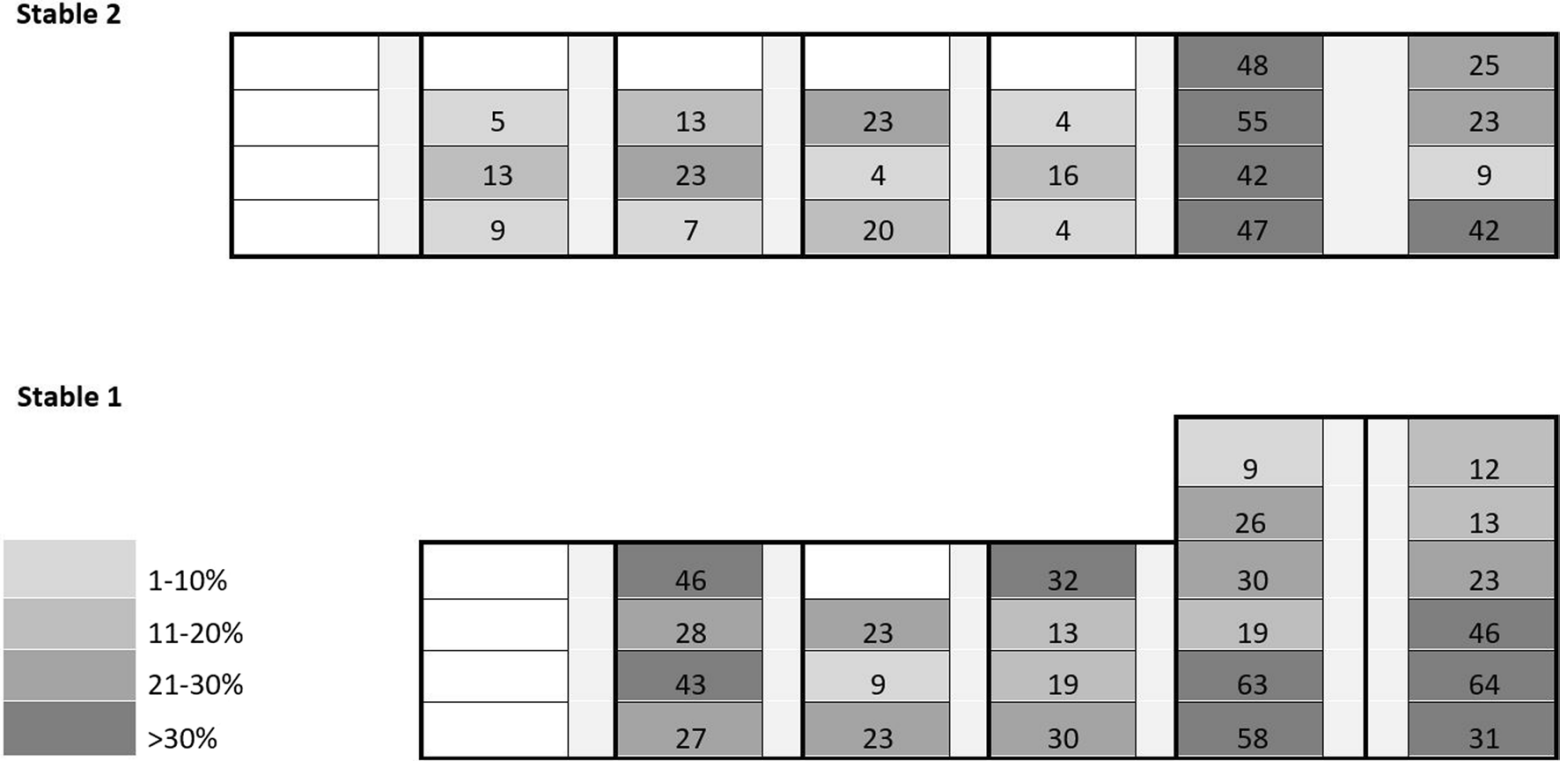
The mean prevalence (%) of porcine ear necrosis for the different pens of the nursery unit at the end of the rearing period. Only pens in which pigs for three weaning batches were housed, were included. An arbitrary color code indicates prevalence groups (light grey 10% or less; dark grey more than 30%)

The prevalence of PEN at the pen level ranged from 7 to 63%. The stable plan showing the location of pens and their average prevalence is shown in Fig. 2. There was no single pen without affected pigs. When assessing the repeatability of PEN prevalence within a pen, the interclass correlation coefficient was 0.09 (*P* > 0.05).

Table 2 shows the number of pigs that were affected unilaterally and bilaterally. Fourteen percent of all pigs had lesions on one ear, while 9% were affected on both ears.

**Table 2:** The percentage (number) of pigs with unilateral or bilateral porcine ear necrosis lesions

Weaning batch	1	2^a^	3^a^	4	5	6^a^	Total
Total number of pigs	583	547	676	672	749	616	3898
% (number) of affected pigs	22 (127)	32 (173)	11 (75)	24 (159)	20 (150)	32 (197)	23.5 (881)
% (number) of pigs affected unilaterally	12 (69)	20 (107)	8 (52)	14 (94)	12 (93)	19 (120)	14.1 (535)
% (number) of pigs affected bilaterally	10 (58)	12 (66)	3 (23)	10 (65)	8 (57)	13 (77)	9.3 (346)
% of pigs affected bilaterally within animals with lesions	46	38	31	40	38	39	39

Data collected at the end of the nursery in the six weaning batches

^a^A mycotoxin detoxifier was added to the feed of weaning batches 2, 3 and 6

### Severity of PEN lesions

The proportion of pigs with different severity lesion scores (1–4) from the different weaning batches at the end of the nursery is shown in Table 3. Within all recorded lesions (n = 1243) the majority had score 1. The prevalence in the different batches ranged from 76.0 to 90.0%. The prevalence of score 2 lesions varied from 8.0 to 21.5%, the prevalence of score 3 from 0.3 to 3.0%. Score 4 lesions only appeared in two pigs. The average percentage of lesions scored as 1, 2, 3, and 4 of all the batches were 84.6%, 14.0%, 1.3% and 0.1%, respectively.

**Table 3:** The percentage of porcine ear necrosis lesions according to severity score (from 1 to 4)

Weaning batch	1	2^a^	3^a^	4	5	6^a^	Total
Total number of lesions^b^	201	239	98	224	207	274	1243
*Percentage distribution of the lesions (%)*							
Score 1	78.0	90.5	76.5	88.0	90.0	84.5	84.6
Score 2	19.0	8.0	21.5	11.5	9.5	14.5	14.0
Score 3	3.0	1.5	2.0	0.5	0.5	0.3	1.3
Score 4	0.0	0.0	0.0	0.0	0.0	0.7	0.1

Data collected at the end of the nursery period in the six weaning batches

^a^A mycotoxin detoxifier was added to the feed of weaning batches 2, 3 and 6

^b^The total number of lesions is the sum of the number of pigs with unilateral lesions and twice the number of pigs with bilateral lesions

### Effect of mycotoxin detoxifier on PEN lesions

There was no significant difference in the percentage of pigs with PEN lesions at the end of the nursery between the weaning batches with or without mycotoxin detoxifier in the feed (22% vs. 25%; *P* > 0.05).

### Average daily gain and mortality

The ADG is shown in Table 4. The mean ADG (± SD) for pigs with (n = 158) and without (n = 243) PEN lesions was 394 g (± 65 g) and 391 g (± 71 g), respectively. Taking into account the effects of batch, pen and feed (use of mycotoxin detoxifier or not), no statistically significant differences were seen between the pigs that had PEN lesions and the ones that did not have PEN lesions (*P* > 0.05). Batch 2 had the highest ADG (430 ± 87 g), while the lowest ADG was observed in batch 5 (372 ± 60 g).

**Table 4:** Average daily gain (g) during the nursery period of a group of pigs (99 to 104 pigs per batch)

Weaning batch	3^a^	4	5	6^a^	Total
Pigs with PEN	378 (35%)	416 (25%)	373 (32%)	404 (66%)	394 (40%)
Pigs without PEN	394 (64%)	415 (75%)	371 (68%)	375 (34%)	391 (60%)
All pigs	388 (n = 99)	415 (n = 100)	372 (n = 104)	394 (n = 98)	392 (n = 401)

There were no statistically significant differences between the ADG of pigs with and without PEN lesions

Only weaning batches 3 to 6 were included, as for these batches, ADG of individual pigs from the subgroups was linked with the presence of porcine ear necrosis (PEN) lesions at the end of the nursery. ^a^A mycotoxin detoxifier was added to the feed of weaning batches 3 and 6

The ADG (± SD) of pigs with PEN lesions on both ears (n = 346), one ear (n = 535) and of non-affected pigs (n = 243) were 399 g (± 70 g), 392 g (± 64 g), and 392 g (± 71 g), respectively (*P* > 0.05).

The ADG (± SD) of pigs with score 2 and 1 were 420 g (± 44 g), 387 g (± 68 g), respectively (*P* > 0.05). The ADG for pigs with score 3 and 4 was not calculated, as there were no weighed pigs with score 4, and only one pig with score 3.

The mortality in batches 1 to 6 was 0.9%, 2.5%, 1.6%, 1.0%, 0.5%, and 2.1%, respectively.

### Mycotoxins in the feed

The concentration of different mycotoxins (min–max) in pooled samples of all weaning batches is shown in Table 5. Type A-trichothecenes (4–84 μg/kg), type B-trichothecenes (78–227 μg/kg), and ergot alkaloids (68–260 μg/kg) were present in all samples. Also enniatin B (9–61 μg/kg) and enniatin B1 (8–56 μg/kg) were found. The concentration of deoxynivalenol (DON) varied from 62 to 175 μg/kg, and the concentration of zearalenone (ZEN) from 2 to 14 μg/kg.

**Table 5:** Concentration of mycotoxins (µg/kg) present in the feed

Mycotoxin	Weaning batch							Average
		1	2^b^	3^b^	4	5	6^b^	
Type A-trichothecenes	A	34/6/11	95/33/22	58/28/84	27/14/3	42/4/40	35/68/37	36
	B	17	50	57	15	29	47	
Type B-trichothecenes	A	227/174/105	274/119/134	133/80/188	88/90/78	81/107/114	110/138/152	133
	B	169	176	133	85	101	133	
Deoxynivalenol^a^	A	175/114/89	136/71/80	131/67/131	75/76/62	62/85/92	72/72/106	94
	B	126	96	110	70	80	83	
Enniatin B	A	30/24/27	61/36/17	37/12/42	23/15/9	15/16/32	14/55/12	26
	B	27	38	30	16	21	27	
Enniatin B1	A	26/24/18	50/24/12	31/10/35	17/10/8	12/14/31	13/56/10	22
	B	22	28	25	12	19	26	
Ergot alkaloids	A	215/260/121	150/96/191	104/227/197	105/220/119	97/68/121	97/98/82	143
	B	199	146	176	148	96	92	
Zearalenone^a^	A	4/6/5	5/6/6	9/3/2	2/2/2	4/4/2	14/2/4	5
	B	5	6	5	2	3	7	
Tenuazonic acid	A	0/0/0	0/0/0	0/0/0	0/56/0	0/0/0	55/53/59	56
	B	0	0	0	56	0	56	
Alternariol-monomethyl ether	A	2.7/0.6/0.9	1.0/0.9/1.4	1.1/1.0/0.5	0.8/0.7/0.6	0.9/0.6/0.6	1.1/0.6/0.4	0.9
	B	1.4	1.1	0.9	0.7	0.7	0.7	

For each weaning batch, three pooled feed samples were collected. The results of the three individual samples (A) are shown as well as the mean of the three pooled samples (B). The last column shows the total average per mycotoxin

^a^EU reference value in feed for pigs and piglets for deoxynivalenol is 900 µg/kg, and for zearalenone 100 µg/kg, respectively

^b^A mycotoxin detoxifier was added to the feed of weaning batches 2, 3 and 6

### Mycotoxins and mycotoxin metabolites in plasma

Mycotoxins and metabolites in the plasma samples that were present in a concentration above the limit of quantification (LOQ) are presented in Table 6. Overall enniatin B, enniatin B1, tenuazonic acid, and alternariol monomethyl ether were present in 21%, 8%, 25%, and 3% of the plasma samples, respectively. Zearalenone and DON were not detected in any plasma samples or were below the LOQ.

**Table 6:** Percentage of plasma samples in which mycotoxins were found above the level of quantification

Mycotoxin	Week	Weaning batch						Total	Average concentration (ng/ml)
		1	2^a^	3^a^	4	5	6^a^		
Enniatin B	4	0	80	60	10	10	60	37	0.085
	7	0	0	0	0	30	0	5	0.056
Enniatin B1	4	0	30	20	0	0	10	10	0.050
	7	0	33	0	0	0	0	6	0.054
Tenuazonic acid	4	0	70	40	40	70	70	48	1.463
	7	0	0	0	10	0	0	2	1.962
Alternariol-monomethyl ether	4	0	0	10	0	0	0	2	0.511
	7	0	22	0	0	0	0	4	1.770

Data presented for each weaning batch and for all weaning batches together, with the average mycotoxin concentration. Fifty-nine animals were blood sampled twice, at 7 and 10 weeks of age (4 and 7 weeks post-weaning, respectively)

^a^A mycotoxin detoxifier was added to the feed of weaning batches 2, 3 and 6

### Serological results

The percentage of pigs with serum antibodies against SI subtypes H1N1, H1N2, and H3N2, PCV2, PRRSv and *M. hyopneumoniae* at 7 and 10 weeks of age are shown in Table 7. The average percentage of pigs with antibodies against H1N1, H1N2 and H3N2 at 7 and 10 weeks of age were 97% and 96%, 33% and 38%, and 23% and 3%, respectively. All sampled animals were seronegative for PCV2 (IgG and IgM) antibodies and *M. hyopneumoniae* at 7 weeks of age, whereas more than 30% and 60% of the pigs had antibodies against PCV2 (IgG and IgM) and *M. hyopneumoniae*, respectively, at 10 weeks of age. The percentage of pigs with antibodies against PRRSv at 7 and 10 weeks of age was 70 and 52%, respectively.

**Table 7:** Percentage of pigs with antibodies in serum

	Weaning batch						
	1	2^a^	3^a^	4	5	6^a^	Total
Nb of sampled animals	(n = 10)	(n = 9)	(n = 10)	(n = 10)	(n = 10)	(n = 10)	(n = 59)
	4 weeks post-weaning (7 week old)						
Influenza subtype	Blood samples not taken						
H1N1				90	100	100	97
H1N2				40	0	60	33
H3N2				10	0	60	23
PCV2 IgG				0	0	0	0
PCV2 IgM				0	0	0	0
PRRSv				70	70	70	70
*M. hyopneumoniae*				0	0	0	0
	7 weeks post-weaning (10 weeks old)						
Influenza subtype							
H1N1	100	77	100	100	100	100	96
H1N2	40	89	70	30	0	0	38
H3N2	0	0	0	10	10	0	3
PCV2 IgG	100	56	70	60	40	60	65
PCV2 IgM	80	67	80	70	50	90	61
PRRSv	50	44	40	50	70	60	52
*M. hyopneumoniae*	50	33	50	50	30	30	40

Serum tested for swine influenza H1N1, H1N2, H3N2, porcine circovirus type 2 (PCV2), porcine reproductive and respiratory syndrome virus (PRRSv) and *Mycoplasma hyopneumoniae* (*M. hyopnemoniae*) at 7 and 10 weeks of age (4 and 7 weeks post-weaning, respectively)

Only pigs from weaning batches 4–6 were sampled twice, at 4 and 7 weeks post-weaning

^a^A mycotoxin detoxifier was added to the feed of weaning batches 2, 3 and 6

### PCR test

Only 2 out of 21 pooled samples were PCR positive for PCV2. One in PEN positive and one in PEN negative animals. The PRRSv EU strain was found in 3 out of 21 pooled samples, all in PEN negative pigs, where the North American PRRSv strain was not found.

### Climate in stables

The average ambient temperature and humidity in batches 4–6 were 26.2 °C and 56.6% (batch 4), 26.0 °C and 59.6% (batch 5) and 26.4 °C and 51.1% (batch 6). Overall, the mean ambient temperature for entire nursery period of these batches was 26.2 °C, and the mean relative humidity 55.7%.

### Water analysis

The results of the drinking water analysis are shown in Table 8. The drinking water contained an orange sediment, and two other parameters were slightly higher than the maximum concentrations proposed by the laboratory namely the number of intestinal enterococci (2 colony forming units (CFU) per 100 ml), and sulfite reducing clostridia (1 CFU per 20 ml). All other parameters were within the range of the reference values.

**Table 8:** Results of bacteriological, biochemical and macroscopic analysis of the drinking water used in both stables

Parameter	Result	Laboratory reference values
*Macroscopic evaluation*		
Physical appearance	Orange sediment	Bright
Smell	No	No
Color	Yellowish	Colorless
*Bacteriological analysis*		
Number of coliforms (cfu/ml)	0	< 100
Intestinal enterococci (cfu/100 ml)	2	< 1
Sulfite-reducing clostridia (cfu/ 20 ml)	1	< 1
Aerobic bacteria (22 °C) in total (cfu/ml)	2400	< 100,000
Aerobic bacteria (37 °C) in total (cfu/ml)	530	< 100,000
*Chemical analysis*		
pH	7.70	4–9
Ammonium (mg/l)	0.78	≤ 2
Nitrates (mg/l)	< 10	≤ 200
Nitrites (mg/l)	< 0.10	≤ 0.5
Sulfates (mg/l)	< 5.0	≤ 250
Total hardness (°D)	13.0	≤ 20
NaCl (mg/l)	26.0	≤ 3000

## Discussion

The present study showed a high prevalence of PEN in nursery pigs in this farm, with almost one out of four pigs (23.5%) being affected at the end of the nursery. The lesions mainly appeared from the fourth week after weaning onwards. The majority of the pigs (85%) had mild lesions and about 40% of the affected piglets had PEN lesions on both ears. The prevalence of PEN was not significantly influenced by the addition of a mycotoxin detoxifier to the feed. The ADG during the nursery period was not lower in affected pigs compared to pigs without PEN lesions.

The high prevalence of PEN in the present study as well as the main onset of the lesions in pigs of approximately 7 weeks of age, are similar to the results reported by Papatsiros [18]. The prevalence, onset and progression of PEN lesions found in the studies by Busch et al. [14, 19] were quite different. In the latter studies (70% [14], 45% [19]), much more piglets were already affected shortly after weaning. Petersen et al. [15] found in a large-scale study in Danish finishing pigs an overall PEN prevalence of 4.4, with PEN being the most prevalent clinical sign. It occurred in 30% of the pigs with clinical signs. Pringle et al. [16] also found a high prevalence of PEN in pigs in organic farms in Sweden, with lesions starting around weaning (6 weeks of age) and reaching prevalence values of 50 to 70% in the nursery during winter period.

The present study also assessed the severity of the PEN lesions. To this end, an arbitrary scoring system was developed that can be used easily in commercial farms. The majority of the affected pigs (85%) had mild lesions, which corresponded to small crusts on the ear tip. Comparing the severity of the PEN lesions with literature data is difficult, as most studies only assessed the prevalence but not the severity of the lesions [15, 16]. The high prevalence of mild lesions indicates that one should look carefully to the ears of the pigs when performing clinical examination. Else only severely affected pigs might be detected and PEN problems in the farm might be underestimated. It was also shown that in most pigs (61%), PEN lesions were present on one ear. This prevalence was consistent among the different weaning batches.

In practice, it is sometimes argued that PEN problems occur more in specific pens or parts of the barn, possibly because the air quality is insufficient in these places. Based on three weaning batches, pigs housed in specific pens of the nursery unit were more affected than in other pens. However, pens with a low (≤ 10%) and high (> 30%) prevalence were scattered across the two stables, and were not present in specific areas of the nursery unit. Also differences in prevalence between pigs of consecutive weaning batches housed in one pen were high. For instance, pens with a high PEN prevalence from one weaning batch could have a low prevalence in the next weaning batch. Logically, the intra class correlation coefficient for PEN prevalence at pen level was low and not statistically significant. This means that in the present farm, specific pens with pigs being at high risk to develop PEN could not be predicted on beforehand.

The results showed that PEN lesions did not decrease the ADG of the piglets in the present farm. Busch et al. [14, 19] also reported that PEN had no effect on the pigs’ growth. In the study of Papatsiros [18] pronounced effects on performance were reported. However, in that study, PCV2 infection causing post-weaning multisystemic wasting syndrome (PMWS) was likely the most important problem in the farm, and PEN might have been secondary, as the PCV2 was detected, there was a high piglet mortality (up to 15%), wasting and poor growth of the piglets as well, which are typical signs for PMWS.

The absence of an effect of PEN on performance in the present study might be due to the fact that most lesions were mild and occurred only in the last few weeks of the nursery period, leaving insufficient time to exert negative effects on performance. However, the absence of a negative effect of score 2 and 3 lesions might be due to the low number of pigs with these scores and also the late onset in the nursery. Further research is warranted to elucidate the effects of more severe lesions and to investigate whether there is a potential negative effect on performance during the fattening period.

The presence of different mycotoxins in the feed was investigated, as mycotoxins have been described as potential risk factors for necrosis [10]. The analysis was based on different samples collected from every weaning batch, as mycotoxins might not be homogeneously distributed in the stored feed [23]. Next, prior to analysis, two successive feed samples from a weaning batch were pooled to one pooled sample. The latter pooling procedure was used mainly to limit the costs for analyses. In the end, three pooled samples of feed from each weaning batch were analyzed.

In the present study, the mycotoxin concentrations in feed were generally low. The average concentrations for DON (94 µg/kg) and ZEN (5 µg/kg) in all samples were below the EU regulatory maximum guidance values [24] for complementary and complete feeding stuffs for piglets (900 and 100 µg/kg, respectively). The maximum concentrations that have been found for the individual feed samples for DON (175 µg/kg) and ZEN (14 µg/kg) were also below the EU maximum guidance values. According to Devreese et al. [25], measuring specific toxins or their metabolites in the blood is an alternative way for testing individual animal’s exposure or to evaluate the efficacy of reduction strategies such as detoxifiers. Enniatin B was the most prevalent mycotoxin in the plasma samples in the present study, and it is also described as the most frequently detected mycotoxin from the enniatin-group in grain in the EU [26]. Enniatin B might promote cell damage and interfere with cell defense mechanisms [27]. The main clinical manifestations attributed to ergot alkaloids intoxication are: decreased animal performance, gangrene with necrosis of the ear or tail, lameness, nervous symptoms, and agalactia [28, 29]. In the current study, the feed alkaloids concentration ranged between 68 and 227 µg/kg, where the study of Oresanya et al. [30] has shown that only weaners fed with feed containing more than 1000 µg/kg manifested decreased average daily gain and feed intake. At the same time, no animal (highest alkaloid concentration in the feed was 20,800 µg/kg) developed nervous signs or cutaneous lesions during the 4 week study time. Further research is needed to assess the importance of these mycotoxins or metabolites, and also to investigate possible additive or synergistic effects between different mycotoxins [31].

There was no significant difference in the PEN prevalence at the end of the nursery in the weaning batches with (22%) or without (25%) the mycotoxin detoxifier added to the feed. This might be due to the low mycotoxin contamination levels in the feed. Also, in general, not all mycotoxins in the feed can be detoxified, and/or some might only be partially detoxified [32]. The plasma levels of respective mycotoxins (DON, ZEN) were below the LOQ, also in batches not receiving the detoxifier. The timing of blood sampling in relation to consumption of contaminated feed may also influence mycotoxin exposure assessment [32]. The maximum plasma concentration for DON and ZEN is reached within 1 h after feed intake in pigs, and these toxins are efficiently eliminated leading to a rather fast decrease in plasma levels [33, 34]. This timespan could not be assessed in the present study as the pigs were fed ad libitum and had permanent access to feed. However, the blood sampling for the different weaning batches was always conducted at a similar time of the day, namely between 10.00 am and 1.00 pm. Therefore, it is unlikely that the time of sampling had biased the comparison of results between different batches.

The fact that mycotoxins were present at low concentrations and that addition of a detoxifier did not change the prevalence, suggests that an internal trigger like mycotoxins was unlikely as primary cause of the problems in the present farm.

Based on the serological results, there was a clear seroconversion between 4 and 7 weeks post-weaning for PCV2 and *M. hyopneumoniae*. However, only two pools of blood samples were positive for PCV2 by PCR. Previous studies [4, 18] have suggested a possible role of PCV2 infections in PEN problems, as vaccination against PCV2 led to a significant improvement of the PEN problems. Whether infections with PCV2 and *M. hyopneumoniae* have contributed to the problem, warrants further study. The seroprevalence of PRRSv declined from 70 to 50% at 4 and 7 weeks post-weaning. Therefore, the involvement of these pathogens in the problem is unlikely. Apart from the hemolytic anemia and mild icterus which may occur after the infection [35], *M. suis* in pigs, similarly to *Mycoplasma pneumoniae* in humans and *Mycoplasma gallisepticum* in chickens, is able to induce the expression of cold-reactive autoantibodies (cold agglutinins) against erythrocytes [36], which in low ambient temperature may lead to red blood cells agglutination, and according to the second hypothesis, a possible occlusion of the small vessels in the ear with subsequent necrosis. Cases of dogs with lesions on extremities due to cold agglutinins disease (CAD) have been already described [37], but in pigs not yet. Another question is whether the temperature in facilities for nursery piglets (24–28 °C) is low enough to induce the agglutination. As the main goal of this study was not to find the etiological factor causing PEN, *M. suis* was not investigated in the blood samples. Microbiological culture of swabs from the lesions for *Staphylococcus* sp. and *Streptococcus* sp. have neither been performed. Nevertheless, future studies dealing with PEN, should consider these pathogens in their analyses.

In terms of the third hypothesis, no problems with ear biting have been noticed. In pens where the pig density was lower than average (because of management reasons), the problem with ear lesions occurred as well.

Although MAMPs have been proposed as a possible factor for Swine Inflammation and Necrosis Syndrome (SINS) [7], its role in PEN is unclear. The swine inflammation and necrosis syndrome manifests itself with lesions on tail, ears, heels and soles, claw coronary bands, teats, navel, and/or vulva of suckling piglets. Therefore the relation to PEN which in fact applies only to the ears of weaners, should be investigated. The focus of this study was exclusively on ear necrosis as other parts of the body were not affected in any batch in the past in that farm. The environmental temperature and relative humidity were acceptable for nursery pigs. Also the fact that affected animals were scattered across different pens and that the prevalence was not predictable based on pen location in the nursery, might indicate that ventilation problems had not likely played a major role in the problem.

## Conclusions

The results showed a high prevalence of PEN in nursery pigs in this farm. The lesions were generally mild and appeared from the fourth week after weaning onwards. About 40% of the affected piglets had PEN lesions on both ears. The prevalence of PEN was not significantly influenced by the addition of a mycotoxin detoxifier to the feed, possibly due to the low mycotoxin contamination levels in the feed. The PEN lesions did not adversely affect the growth of the piglets, likely because lesions were mild and occurred during the last weeks of the nursery. Further research is warranted to elucidate the main causes of PEN and to assess the impact of more severe lesions on performance.

## Data Availability

The datasets used and/or analyzed during the current study are available from the corresponding author on reasonable request.

## Footnotes

1 All toxins included in the method: 3-acetyldeoxynivalenol, 15-acetyldeoxynivalenol, alternariol, alternariol monomethyl ether, aflatoxins: B1,B2,M2,G1,G2,M1, beauvericin, de-epoxy-deoxynivalenol, deoxynivalenol, deoxynivalenol-glucuronide, deoxynivalenol-sulfate, enniatins: A,A1,B,B1, fumonisin: B1,B2,B3, HT-2 toxin, ochratoxin A, tenuazonic acid, T2 toxin, zearalanone, α-zearalanol, β-zearalanol, α-zearalenol, β-zearalenol, α-zearalenol-glucuronide, β-zearalenol-glucuronide, zearalenone, zearalenone-glucuronide and zearalenone-sulfate.
